# Stable eye versus mouth preference in a live speech-processing task

**DOI:** 10.1038/s41598-023-40017-8

**Published:** 2023-08-08

**Authors:** Charlotte Viktorsson, Niilo V. Valtakari, Terje Falck-Ytter, Ignace T. C. Hooge, Maja Rudling, Roy S. Hessels

**Affiliations:** 1 Development and Neurodiversity Lab, Department of Psychology, Uppsala University, Uppsala, Sweden; 2 Experimental Psychology, Helmholtz Institute, Utrecht University, Utrecht, The Netherlands; 3 Center of Neurodevelopmental Disorders (KIND), Division of Neuropsychiatry, Department of Women’s and Children’s Health, Karolinska Institutet, Stockholm, Sweden

**Keywords:** Psychology, Human behaviour

## Abstract

Looking at the mouth region is thought to be a useful strategy for speech-perception tasks. The tendency to look at the eyes versus the mouth of another person during speech processing has thus far mainly been studied using screen-based paradigms. In this study, we estimated the eye-mouth-index (EMI) of 38 adult participants in a live setting. Participants were seated across the table from an experimenter, who read sentences out loud for the participant to remember in both a familiar (English) and unfamiliar (Finnish) language. No statistically significant difference in the EMI between the familiar and the unfamiliar languages was observed. Total relative looking time at the mouth also did not predict the number of correctly identified sentences. Instead, we found that the EMI was higher during an instruction phase than during the speech-processing task. Moreover, we observed high intra-individual correlations in the EMI across the languages and different phases of the experiment. We conclude that there are stable individual differences in looking at the eyes versus the mouth of another person. Furthermore, this behavior appears to be flexible and dependent on the requirements of the situation (speech processing or not).

## Introduction

Gaze to faces seems to be an important facilitator of language acquisition^1^, perhaps because the processing of both visual and auditory signals produces a perceptually more salient signal^2,3^. The pattern in which people look at the faces of others varies between individuals, but also depends on a variety of external factors such as social context and the task at hand^4^. When asked to merely listen to a talker, most adults tend to look primarily at the eyes regardless of whether the language is familiar or not^5^. Similarly, Foulsham and Sanderson ^6^ found that adults mainly look at the eyes of a person in a dynamic social scene, regardless of whether the video was muted or not. However, when speech cues are relevant for the task, gaze to the mouth increases^7–10^. For example, Vatikiotis-Bateson, Eigsti, Yano, and Munhall^11^ showed that when viewing videotapes of speakers having monologues, observers looked more at the mouth during high-noise conditions than during low-noise conditions (55% vs 35% of the time, respectively). Yi, Wong, and Eizenman^12^ found that when observing a video of a speaker, performance on a word-recognition task was similar across varying speech-signal-to-noise ratios as long as the observer fixated on a point within 10 degrees of visual angle from the center of the speaker’s mouth. As speech-signal-to-noise ratio was increased, however, observers made more looks to areas closer to the speaker’s mouth. Barenholtz, Mavica, and Lewkowicz^13^ found that monolingual adults’ gaze to the mouth increased when listening to an unfamiliar language when they were given a task that required active speech processing (participants were presented with an audio file after having seen videos containing either familiar or unfamiliar languages and were asked to choose which video the audio file belonged to). When instructed to simply passively watch and listen to the speaker (i.e., in the absence of an explicit speech-processing task), participants gazed equally to the eyes and mouth in response to both familiar and unfamiliar languages. Taken together (and assuming that gaze direction indicates the focus of attention), these studies demonstrate that, in the presence of an explicit speech-processing task, both language familiarity and noise level seem to modulate selective attention to the audiovisual speech cues in a talker’s mouth in adults.

It is possible that changing one’s gaze direction from the eyes to the mouth, when the situation demands it, reflects a general encoding strategy that is present already in early infancy. As shown by Lewkowicz and Hansen-Tift^5^, there appears to be a shift in the pattern of looking at the eyes versus the mouth of a speaker between 4 and 12 months of age: at 4 months of age, infants look more at the eyes than the mouth of a speaker, while at 8–10 months, the mouth is looked at more than the eyes. At 12 months of age, the pattern seems to start shifting back, as infants no longer look more at the mouth than the eyes when hearing their native language^5^. When the language is unfamiliar, however, the shift toward more looking at the mouth seems to persist longer, as 12-month-old infants still selectively look more at the mouth when hearing a nonnative language^5^. Because the eyes and mouth convey slightly different information, individual differences in the tendency to look at either the eyes or the mouth may influence what information is processed, and might shape development over time^14^. The distribution of gaze to the eyes and mouth can be operationalized using the eye-mouth-index (EMI; a measure using the relative dwell time at the eyes versus mouth, resulting in a preference for one of them, regardless of total dwell time at the face). Already at 5 months of age, substantial and stable individual differences in the EMI have been found (across videos of a woman singing, talking, and smiling^14^). Using twin modeling, the EMI at this age was found to be highly heritable. Later in development, at 18–30 months of age, the EMI is stable across conditions consisting of dynamic videos of single persons and multiple people^15^, and eye and mouth looking have been found to be highly heritable at this age as well^16^. However, research on whether these substantial and stable individual differences in the EMI persist into adulthood has been scarce. Studies on face recognition have shown that when identifying faces, there is considerable individual variability in which facial features adults fixate most; for example, some may tend to look at the eyes while some tend to look at the nose^17,18^. These patterns have been shown to be stable over time and unrelated to face recognition performance^19,20^. While the mouth region is strongly associated with visual speech information^21^, the eye region appears to be important for e.g., face recognition^22,23^. Stable individual differences in the tendency to look at either the eyes or the mouth may therefore explain how small behavioral and attentional differences can lead to different developmental trajectories and shape human perception and communication. It is therefore important to study variation in the EMI in adulthood, as well as potential links between the EMI and other abilities, such as language acquisition.

All of the above-mentioned studies of looking behavior used screen-based paradigms to explore gaze in response to both dynamic and static social stimuli. It is typically assumed that the gaze patterns obtained using screen-based paradigms generalize to real interactions (e.g., face-to-face conversations), but to the best of our knowledge, no one has investigated gaze behavior during speech processing in adulthood during a *live* face-to-face interaction between two people, and thus we do not know whether this assumption holds. In this study, we analyzed the gaze of adult participants when hearing a familiar and an unfamiliar language spoken by an experimenter sitting in front of them, while being asked to perform a memory task. We used the eye-mouth-index as our primary measure (scale 0–1, where 0 means looking at the mouth 100% of the time and 1 means looking at the eyes 100% of the time). Based on the findings by Barenholtz, Mavica, and Lewkowicz^13^, we hypothesized that relative time spent looking at the mouth of the experimenter would be longer when listening to an unfamiliar language (Finnish) as compared to when listening to a familiar language (English). We also explored whether relative time spent looking at the mouth predicted the number of sentences the participant remembered (from the familiar language and unfamiliar language, respectively). These hypotheses were pre-registered at OSF (https://osf.io/5fzha/).

## Methods

### Participants

In total, 41 students at Utrecht University completed the experiment. According to self-reports, they were all fluent in English and unfamiliar with Finnish. Three participants were subsequently excluded due to looking at the face of the experimenter less than 25% of the time in each condition, leading to a final sample of 38 participants (see “Eye-tracking data quality and exclusion” for details). Twenty-eight of these were females (73.7%), and the mean age was 23.97 years (min = 19 years, max = 31 years). The first languages of the participants were as follows (number of participants shown in parentheses): Albanian (1), Arabic (1), Cantonese (1), Catalan (1), Chinese (2), Dutch (21), English (1), French (1), Galician (1), German (2), Greek (3), Italian (1), Mandarin (2).

This research project does not belong to the regimen of the Dutch Act on Medical Research Involving Human Subjects, and therefore there was no need for approval of a Medical Ethics Committee. However, following institutional guidelines, the study was approved by the Ethics Committee of the Faculty of Social and Behavioural Sciences of Utrecht University (protocol nr. 22-0251). Informed consent was collected from all participants.

### Apparatus

**Figure 1:**
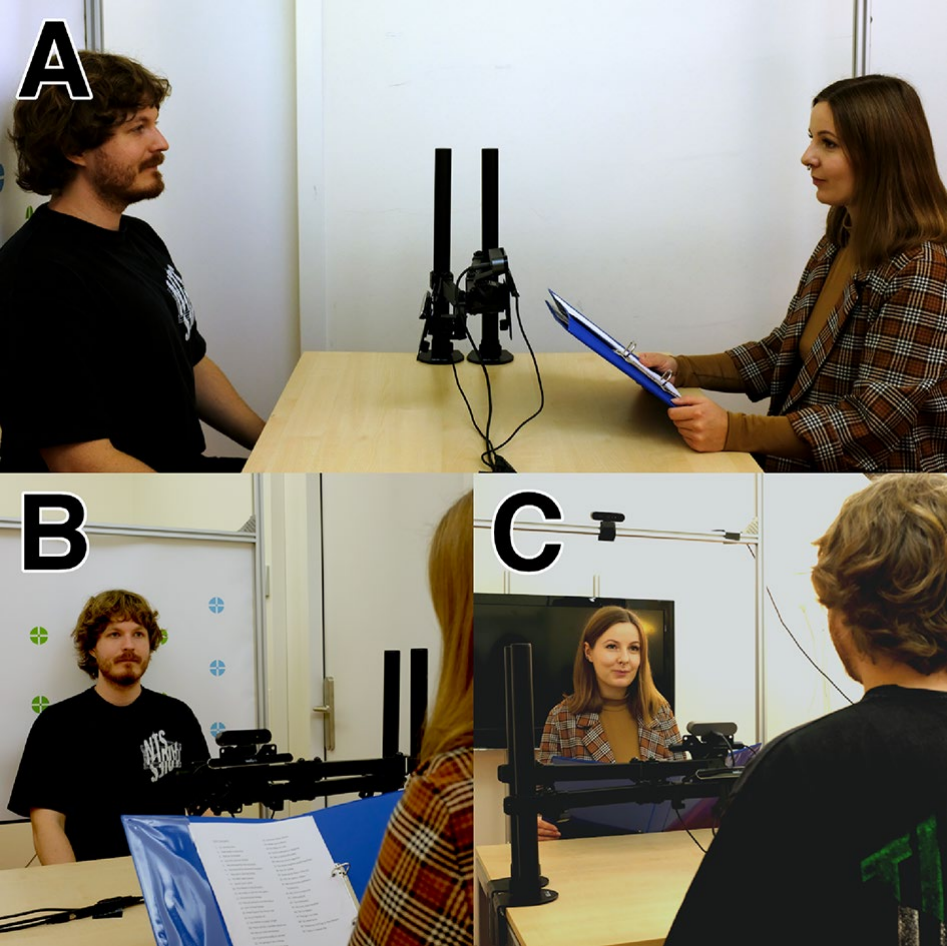
An illustration of the setup from (**A**) a side view, (**B**) the view of the experimenter, and (**C**) the view of the participant. The participant and experimenter sat across each other on a table (depicted here are authors CV and NV). Two Tobii Pro Nano remote eye trackers mounted on monitor arms attached to the table were used to simultaneously record the gaze position of both. One Logitech BRIO web camera mounted on the monitor arm facing the experimenter (visible in all panels) was used to record the scene in front of the participant. Another Logitech BRIO web camera mounted on the aluminum frame behind the experimenter (visible in panel **C**) was used to record the scene in front of the experimenter. A movable TV screen (visible in panel **C**) was used to aid the calibration of the eye tracker on the participant’s side, while a poster with calibration stimuli attached to the aluminum frame behind the participant (visible in panel **B**) was used to aid calibration of the eye tracker on the side of the experimenter.

For our research questions, it was essential that the setup allowed for face-to-face interaction while recording the gaze of both participant and experimenter. We further needed to be able to distinguish which facial features of the experimenter the participant looked at with sufficient accuracy and precision. Hence, we opted for a head-boxed dual eye-tracking setup without screens^24^. Two Tobii Pro Nano 60 Hz eye trackers were used to simultaneously record the gaze of the participant and experimenter. The Tobii Pro Nano is a video-based remote eye tracker using the pupil- and corneal reflection technique. The eye tracker recording the gaze of the experimenter was used to only validate the experimental procedure in which the experimenter looked up at the face of the participant every three sentences (see below). Two Logitech BRIO web cameras (recording at a resolution of 1920 by 1080 pixels and a frequency of 30 Hz) were used as scene cameras to provide a view of the scene in front of both experimenter and participant. The two eye trackers and two web cameras were controlled from one operating computer running Windows 10. This was done to facilitate synchronization of the eye tracker and web camera streams. The participant and experimenter sat across each other on opposite sides of a table with the eye trackers mounted on monitor arms in between. The web camera filming the scene in front of the participant was also mounted on one of the monitor arms, while the web camera filming the scene of the experimenter was mounted on top of an aluminum frame placed behind the participant. A movable TV screen was used to aid the calibration of the eye tracker on the participant’s side, while a poster with calibration stimuli attached to the aluminum frame behind the participant was used to aid calibration of the eye tracker on the side of the experimenter. The configuration of the setup is illustrated in Fig. 1.

### Experimental procedure

The participant was seated on one side of a table with an experimenter seated across them on the other side. The experimenter was the same for each measurement. At the start of each measurement, both the experimenter and participant first completed a calibration and validation procedure (explained further in the “From raw gaze coordinates to gaze coordinates in the world” section below). Following, the experimenter informed the participant that two lists of sentences would be read out loud to them, one in English and one in Finnish, and that they should do their best to remember them. The participant was further told that afterwards a new list would be read to them, and they would be asked to determine whether those sentences had been read before or not. The first list contained 25 English sentences (the familiar language), such as “Today is a great day” and “I’m going to see a movie”. The second list contained the same 25 sentences translated to Finnish (the unfamiliar language), with slight adjustments in order to create sentences with the same number of syllables (see Supplementary Information S1 for the complete lists). The order of the lists was counterbalanced, with 21 participants hearing the English sentences first and 20 participants hearing the Finnish sentences first. The experimenter read the sentences out loud as similarly as possible to all participants. To further minimize variation in experimenter behavior, she looked up at the face of the participant only every third sentence (determined to be an amount that did not feel too excessive based on pilot experiments). For the remaining time, she looked down at the paper where the sentences were written. After both lists of sentences had been read out loud, the participant was asked to complete a final validation procedure by looking at the eyes and mouth of the experimenter in a fixed order. Eye tracking and video data was only recorded up to this point. Next, the experimenter read a new list of 50 sentences (half of them in English and half of them in Finnish, in a mixed order). When reading this list, the experimenter looked down at the paper during every sentence. Thirty sentences had been read to the participant in the first part of the session and twenty sentences were new to them. After each sentence, the participant was asked to say yes or no, depending on whether they believed the sentence had been read to them before or not. Each participant answer was marked down on a sheet of paper by the experimenter.

### Processing the eye tracker signal

#### From raw gaze coordinates to gaze coordinates in the world

To know where the participant and experimenter looked in the scenes in front of them, the raw gaze coordinates provided by the eye trackers needed to first be related to pixel coordinates on the scene camera recordings. This was done by conducting a self-built calibration procedure. This procedure consisted of two parts. The first part was conducted prior to each recording, when the participant was present. In this part, the participant was asked to look at a set of five spinning spirals on a movable TV screen placed where the experimenter would sit during the experiment (see Fig. 1C), while the experimenter was asked to look at a set of four calibration points printed on a poster attached to the aluminum frame behind the participant (see Fig. 1B). Directly after viewing the calibration stimuli, both participant and experimenter looked at a separate set of an equal number of validation stimuli used to later assess data quality. The second part was conducted offline, after each recording, before any further processing of the eye-tracking data. In this part, the pixel positions of the calibration stimuli as they appeared on the scene camera recordings along with the median gaze position from when the experimenter or participant was looking at those stimuli were fed to an optimization algorithm (the *fmin* function in the ‘optimize’ module of the SciPy Python library), allowing us to transform the raw gaze coordinates to pixel coordinates on the scene camera recordings. Next, the gaze position signal was downsampled to match the 30 Hz recording frequency of the web cameras and each frame in the scene camera recordings was assigned its corresponding gaze position coordinate.

#### Constructing areas of interest and calculating gaze-based measures

**Figure 2:**
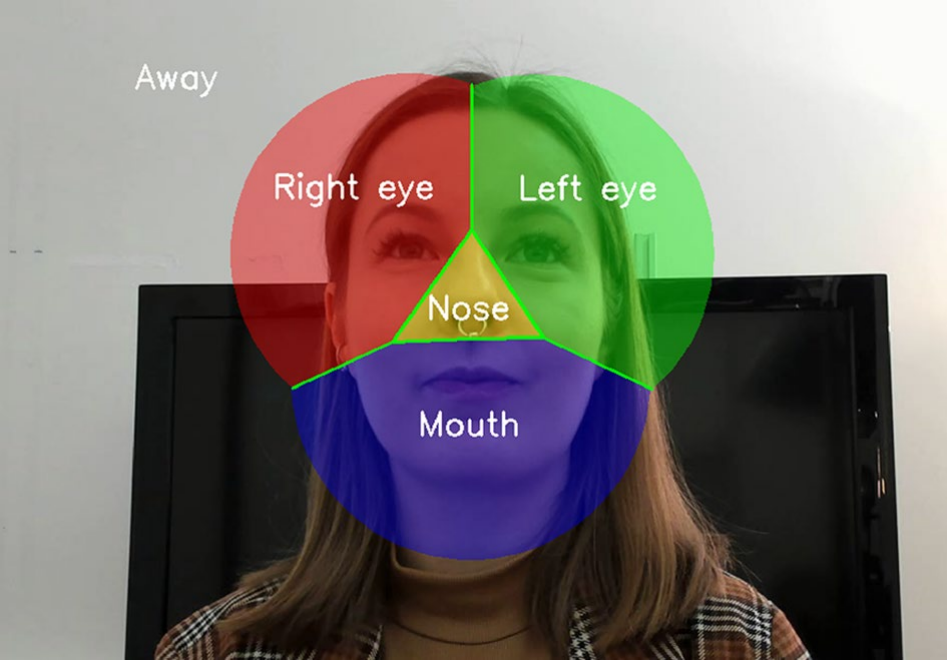
An illustration of the AOI assignment procedure. The colors represent the left eye, right eye, nose, and mouth AOIs. For our analysis purposes, the left and right eye-AOIs were combined into a single eyes-AOI. If the gaze point was within the borders of an AOI, it was assigned to that AOI. If the gaze point was not on any AOI, it was assigned to an away-AOI.

For area of interest (AOI) construction and assignment, we follow the procedure introduced in Hessels et al.^25^. The latest OpenFace version (2.0^26^) was first used to automatically determine the pixel coordinates of specific facial landmarks, which were then used to construct the relevant AOIs: the left eye, right eye, nose, and mouth. For each frame of each scene camera recording for each participant, we then knew both the estimated gaze point of the participant and the locations of the AOI centers on the face of the experimenter (and vice versa), all in pixel coordinates. The gaze coordinate for each frame was assigned to the AOI it was closest to the center of. If the closest distance exceeded two times the mean AOI span for that participant (i.e., the average distance between each AOI center and its closest neighboring AOI center^25^), gaze was assigned to an away-AOI (see Fig. 2 for an illustration of the AOI separation). This method is known as the Limited-Radius Voronoi Tesselation method. It has been extensively validated for face stimuli^27^. Next, we computed the eye-mouth-index (EMI) for each participant, separately for each condition. The EMI was calculated by dividing the combined total dwell time on both eye-AOIs by the combined total dwell time on both eye-AOIs and the mouth-AOI. An EMI value of one thus indicates that the participant looked only at the eyes of the experimenter while an EMI of zero indicates that they only looked at the mouth, out of all time spent looking at both the eyes and the mouth. Lastly, we computed total experimenter dwell time on the participant’s face for each condition by summing the total dwell time on the eyes, nose, and mouth of the participant in seconds.

#### Minimizing parallax error

With scene-camera based eye tracking, one commonly encountered problem is parallax error^24^. In practice, this means that if the participant being looked is not positioned exactly in the same 2D plane the eye tracker was calibrated in, there will be a systematic offset in the reported gaze position. For example, when looking at one’s eyes, the reported gaze position may be somewhere on the forehead instead. We attempted to minimize parallax error for the participant’s gaze signal by calibrating the eye tracker recording their gaze using a movable TV screen positioned as close as possible to where the experimenter would later sit. Despite this, we still observed a small shift due to parallax error in the gaze position signal for most participants. For 27 of our participants, correcting for this was relatively straightforward, as we had conducted an extra validation procedure at the end of the measurement by having participants look at the right eye, left eye, and mouth of the experimenter (see Supplementary Information Fig. S2). The gaze position signal of those participants was shifted a fixed number of pixels by individually examining how much the reported gaze position during the extra validation procedure deviated from the position of those facial features as they appeared on the video frame. For the remaining 14 participants, the magnitude of the shift was determined by first plotting the full gaze position signal over both conditions on top of one frame of the scene camera video where the face of the experimenter was upright and fully visible (see Supplementary Information Fig. S3). The cluster of gaze position coordinates closest to the face of the experimenter was then shifted to match the location of the experimenter’s face as it appeared on the video frame, keeping in mind that the experimenter’s head orientation would change as she looked down at the sentences. Following, we randomly sampled a set of five measurements separately from the participant pools both with and without the extra validation procedure. We then further overlayed the adjusted gaze position signal of those participants on their respective scene camera recordings and visually examined them to confirm that AOI assignment was sufficiently reliable. Parallax error in the gaze position signal of the experimenter was corrected for and checked for reliability in the same way as with the participants without the extra validation procedure.

### Statistical analyses

Differences in the variables between conditions and different phases of the experiment were analyzed using paired-samples t-tests. Associations in the variables between conditions and different phases of the experiment were assessed using Pearson’s correlation.

## Results

### Eye-tracking data quality and exclusion

Before running any analyses, we first ensured that our eye-tracking data quality was sufficient to reliably map the gaze position data to our AOIs (for reporting data quality, we follow the guidelines by Holmqvist et al.^28^). We computed the average validation accuracy, precision, and data loss of the gaze position signal of each participant. Accuracy was operationalized by the Euclidean distance from the reported gaze position to the center of its respective validation stimulus in the scene camera image. Precision was operationalized by the root mean square sample-to-sample deviation of the gaze position signal. Values for accuracy and precision are reported in degrees, assuming a fixed distance of 132 cm from the eyes of the participant to the calibrated plane. Mean accuracy across participants was 0.81° (SD = 0.3°) and mean precision was 0.15° (SD = 0.17°). To determine whether accuracy and precision were sufficient for our purposes, we needed to compare them to the AOI span. Since the same person always acted as the experimenter and was seated in the same position, there was little variation in the AOI span across participants (mean = 2.15°, SD = 0.06°). For most participants, the values for both accuracy and precision were much smaller than the average AOI span, suggesting reliable AOI assignment. For the participants with the worst values for accuracy and precision, we further visually inspected the gaze position signal superimposed on its corresponding scene-camera recording. Even in the worst cases, AOI assignment seemed reliable. Thus, we deemed the accuracy and precision of all participants to be sufficient for the purposes of our analyses.

The mean percentage of data loss during the validation procedure across participants was 0.21% (SD = 0.41%). As data loss during the validation procedure was extremely low, we assume data loss during the measurements to have occurred mainly due to behavioral reasons (e.g., looking outside of the recording range of the eye tracker). Data loss during the experiment was therefore taken to mean that the participant was not looking at the face of the experimenter and was not used as an exclusion criterion. No participants were excluded from the analyses for data quality reasons. However, to calculate EMI it was important that participants looked at the face of the experimenter at least for a certain amount, which we decided to be 25%. Three participants were subsequently excluded from all the following analyses due to looking at the face of the experimenter for less than 25% of the trial duration (i.e., the time between the beginning of the first sentence and the end of the last sentence) in each condition, leading to a final sample of 38 participants.

For the experimenter, average validation accuracy was 0.77° (SD = 0.19°), precision was 0.12° (SD = 0.05°), and data loss during the validation procedure was 0.43% (SD = 0.91%). We consider these values more than sufficient for reliably computing total experimenter gaze at the participant’s face.

### Eye-mouth-index

There was no difference in the total dwell time at the experimenter’s face between the Unfamiliar (Finnish) and Familiar (English) condition (t(37) = −1.278, p = 0.209; see Table 1 for descriptive statistics). There were considerable individual differences in the EMI in each condition (see Supplementary Information Fig. S1 for distributional plots). On a group level, the EMI was lower (i.e., more overall looking at the mouth) during the Unfamiliar condition (mean = 0.318) compared to the Familiar condition (mean = 0.367), but this difference was not statistically significant (t(37) = 1.939, p = 0.060, Cohen’s d = 0.314). Thus, the participants did not look significantly more at the mouth when hearing the unfamiliar language compared to the familiar language. Individual differences in the EMI were stable across conditions (r = 0.855, p < 0.001), and we found no evidence for an association with trial duration in either the Unfamiliar condition (r = −0.111, p = 0.507) or the Familiar condition (r = −0.227, p = 0.171). In addition, there was no evidence that the total amount of time the experimenter looked at the face of the participant was related to EMI in either the Unfamiliar condition (r = −0.142, p = 0.396) or the Familiar condition (r = 0.038. p = 0.823), suggesting that the experimenter did not influence the participants’ tendency to look at either the eyes or the mouth.

**Table 1:** Descriptive statistics.

	Mean (SD) (Min–max)	
	Familiar condition	Unfamiliar condition
Number of correctly identified sentences	19.42 (2.41) [14–24]	15.08 (2.51) [11–22]
Eye-mouth-index (EMI)	0.367 (0.298) [0.000–0.973]	0.318 (0.267) [0.000–0.973]
Total looking time at mouth (s)	23.65 (16.46) [0.90–62.54]	27.55 (16.67) [0.85–60.91]
Total looking time at eyes (s)	11.63 (10.18) [0.00–35.74]	11.48 (9.76) [0.00–35.01]
Total looking time at face (s)^a^	48.03 (14.42) [15.94–68.05]	50.56 (15.29) [21.63–71.05]
Trial duration (s)	64.98 (3.38) [59.84–74.41]	68.45 (3.14) [61.70–74.46]
Total looking time at participant (s)	7.60 (1.21) [5.63–10.96]	8.17 (1.34) [4.81–10.93]

^a^A combined score of aggregated looking time at the eyes, nose, and mouth AOI.

**Figure 3:**
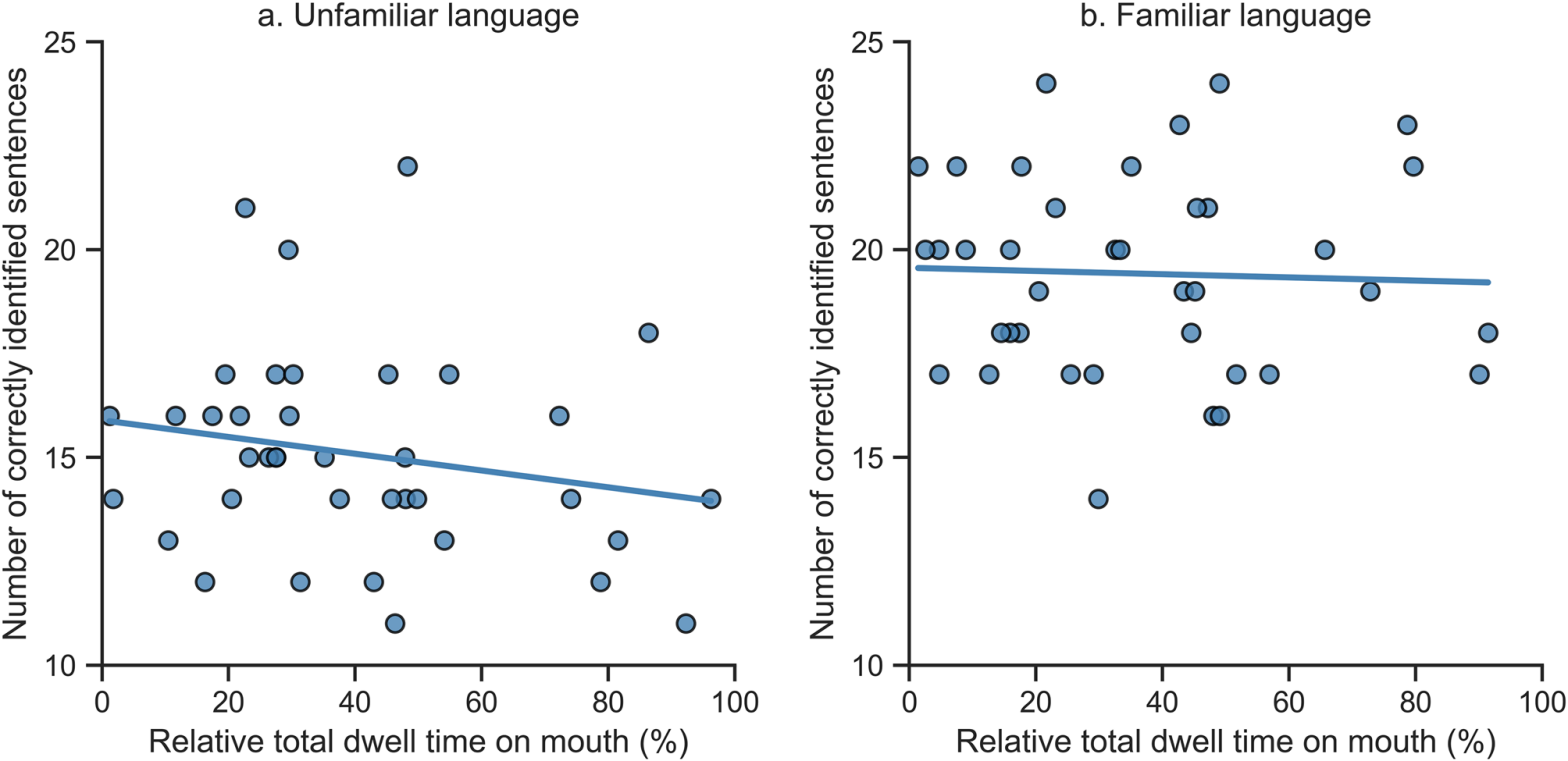
Scatterplots of the association between total percentage of dwell time at the mouth (relative to the total duration of the condition) and number of correctly identified sentences (out of a total of 25 sentences) for the (**a**) Unfamiliar condition and (**b**) Familiar condition.

Participants could correctly identify more sentences in the Familiar condition (mean = 19.42) than in the Unfamiliar condition (mean = 15.08; t(37) = 9.07, p < 0.001). We found no association between the number of correctly identified sentences and trial duration in either the Unfamiliar condition (r = 0.220, p = 0.183) or the Familiar condition (r = 0.038, p = 0.821). In addition, we found no relation between the experimenter's total dwell time at the face of the participant and the number of correctly identified sentences in either the Unfamiliar condition (r = −0.108, p = 0.517) or the Familiar condition (r = 0.208, p = 0.211). Finally, we did not find any relation between the total percentage of looking time at the mouth (relative to the total duration of the condition) and the number of correctly identified sentences in either the Unfamiliar condition (F(1) = 1.496, p = 0.229) or the Familiar condition (F(1) = 0.056, p = 0.814), see Fig. 3.

### Exploratory analyses

Due to the finding that the EMI was stable across conditions, we wanted to explore whether the stability extended to a situation that resembles an instructional conversation and that does not include a specific speech-processing task. The following analyses were not pre-registered. First, we calculated the EMI during a time period of 25 s before the first trial started, where the experimenter explained the test procedure to the participant. We then compared the EMI during the 25-s explanation period to the EMI during each condition. There was a statistically significant difference between the EMI before the experiment started (mean = 0.468) and the EMI during the Unfamiliar condition (mean = 0.318; t(37) = 3.465, p = 0.001) as well as the EMI during the Familiar condition (mean = 0.367; t(37) = 2.451, p = 0.019), meaning that relative looking time at the mouth increased during the speech-processing task. However, the correlation between the EMI before the experiment started and the EMI during the experiment was strong and statistically significant for both the Familiar condition (r = 0.612, p < 0.001) and the Unfamiliar condition (r = 0.517, p < 0.001). Considering the finding that relative total dwell time at the mouth was higher during the speech-processing task than during the instructional part, we wanted to explore whether this shift in gaze allocation might be an effective memory strategy. A difference score was therefore calculated between the EMI during the instructional part and the EMI during each language condition, indicating how much more (or less) the participants looked at the mouth during the sentence-recognition task than during the general instructions. Using linear regression, we found that the difference score did not predict the number of correctly identified sentences in either the Unfamiliar condition (F(1) = 2.449, p = 0.126) or the Familiar condition (F(1) = 0.748, p = 0.393).

## Discussion

In this study, we had adult participants complete a speech-processing task in a face-to-face setting with a physically present experimenter. We examined participants’ gaze behavior using the eye-mouth-index (EMI) while they listened to a set of familiar- and unfamiliar-language sentences they were asked to remember. We did not find significant differences in the EMI between the two language conditions. Instead, we found a strong correlation in the EMI between the conditions and across qualitatively different phases of the experiment/interaction, suggesting stable individual differences in the relative looking time to the eyes versus the mouth.

In contrast to earlier studies of face looking during language processing (e.g.,^13^), we used a novel live dual eye-tracking setup, where the speaker is physically present. This is important as it is the first step to examining whether results from previous screen-based experiments generalize to face-to-face situations where the experimenter is physically present. Notably, we did not find a significant difference in the EMI between the conditions in this live setting, while studies using screen-based paradigms did^13^. This suggests that the results by Barenholtz et al.^13^, who found that adults’ gaze to the mouth increased in response to an unfamiliar language when the task required speech processing, do not generalize to a live, face-to-face setting. Perhaps the physical presence of the experimenter in our study activated social processes that motivated the participants to engage in eye contact, a motivation that was stronger than the incentive to look at the mouth when hearing an unfamiliar language. Another, not mutually exclusive, explanation is that participants were able to gather enough information from the mouth of the experimenter even without looking at it directly. As Yi et al.^12^ have shown, speech remains intelligible as long as one looks within 10 degrees of the mouth (at a distance of approximately 65 cm) even in moderately noisy settings. The experimental setting of the present study was low in noise. It may therefore be that participants did not need to look at the mouth to increase the intelligibility of the unfamiliar sentences, resulting in no difference in the EMI. It is worth noting that the p-value of the analysis of mean EMI differences between conditions was close to significance (0.06). It is possible that a significant difference would have been observed in a larger sample. It is therefore important to interpret our conclusions cautiously before similar studies have been conducted with larger groups and in other settings.

Although we did not find a statistically significant difference in the EMI across conditions, we did find a slight tendency to look more at the mouth than the eyes when the difficulty of the task increased (i.e., from instructions to memory task, and from English to Finnish sentences). This is in line with earlier research showing that visual cues accompanying speech become relevant when difficulty increases (e.g., in a noisy room^7^). Despite this, however, individual differences in the EMI were stable and total looking time at the mouth was not associated with the number of sentences remembered in any of the conditions. This suggests that while switching gaze from the eyes to the mouth might be a common strategy to better understand what is being said, the task was not perceptually constrained to the extent that individual differences could no longer be observed (i.e. the task was not such that one had to look at the mouth to perform it).

The high correlation of the EMI between the conditions in this study shows that adults engaged in a live speech-processing task tend to have a stable relative looking time at the eyes versus the mouth regardless of whether the language of the sentences is familiar to them. This stability is further reinforced by the finding that the EMI was not influenced by trial length or the length of time the experimenter looked at the face of the participant (generally, the experimenter looked up at the participant every three sentences, but with slight variation). Similar results have been shown by Hessels, Holleman, Kingstone, Hooge, and Kemner^29^, who had a confederate either look toward or away from the participant’s face and found that this manipulation did not affect aggregate measures of the participant’s gaze behavior. Stability in which features of a face or body an individual tends to look at has also been shown in other contexts. For example, in face recognition tasks, there are stable individual differences in where people fixate on the face of another, and these differences have been shown to not affect recognition performance^19,20^. In the context of brief social encounters, it has been shown that where one looks on the body of another person depends on the behavior of the person, and, on the individual doing the looking^30^. Thus, gaze behavior can be described by both overall perceptual strategies and stable inter-individual gaze patterns. Given the results of the abovementioned studies, the findings of the present study, as well as studies showing that the EMI is stable in infants^14^ and toddlers^15^, it may be that the general tendency to look at the eyes relative to the mouth is consistent within individuals, throughout life, and across different situations. Further longitudinal studies investigating the EMI in childhood and adulthood are needed to corroborate this, in addition to more extensive cross-situation comparisons.

The findings that the EMI was significantly different but also highly correlated across qualitatively different phases of the interaction between the experimenter and participant further suggest that although the EMI is highly stable on the level of the individual, it is also flexible and dependent on context. This idea is in line with the dynamic systems approach to gaze in face-to-face interaction, which states that interactions can be characterized by sub-states specific to the interactor, the content of the interaction, and the context in which the interaction occurs^4^.

Individual variability and stability in the EMI may have important implications. Visual information regarding the focus of attention, speech, or emotions may be found in multiple areas of the face or body of another person. For example, the results of a multitude of studies indicate that directional cues from the eye region, the head, and the body (i.e., pointing gestures) are used when inferring the focus of attention of another person^31^. Moreover, although the mouth and its surrounding areas have been shown to be strongly associated with visual cues related to speech information^21^, the upper region of the face (i.e., the eyes and forehead) has also been shown to contain cues related to intonation^9^. Finally, information regarding emotional expressions may be contained in multiple areas of the face^32^ and different areas of the face are looked at more when recognizing different emotions^33^. This leads to an important question: whether stable individual differences in the EMI in fact relate to better attunement to specific visual information and whether better attunement to specific visual information may subsequently affect social interaction and perception across development.

### Limitations and future considerations

The design of this study made it possible to examine gaze behavior to the eyes and mouth in a live, face-to-face setting. However, there are limitations to the conclusions that can be drawn. The behavior of both participant and experimenter was restricted by the nature of the task. Our results may shed light on how people look at the face of another during an explicit face-to-face speech-processing task, but we do not know if they can be generalized to more interactive situations. Future studies should focus on incorporating conversations that resemble social interaction in everyday life, as well as more diverse conditions, including different behaviors of the experimenter (such as gestures and varying gaze behavior) and additional levels of difficulty (for example, more difficult speech-processing tasks). It is also important to look at individual gaze shifts in relation to speech processing, rather than just aggregated looking time at the eyes and mouth. For example, gaze to the mouth may be beneficial for speech perception only in situations where speech is ambiguous. In order to elucidate the specific properties of the EMI across different social situations, the temporal aspect of gaze patterns during natural conversation could be considered. Although all participants in this study reported that they speak English fluently, the diverse language backgrounds might have affected the way participants looked at the eyes and mouth. It is therefore important to replicate this study in other cultures and regions, preferably in a way that makes comparisons between language groups possible.

## Conclusions

When an individual is engaged in a live speech-processing task, there is a stable overall trend in how much one looks at the eyes versus the mouth of another person. Furthermore, this behavior appears to be flexible and dependent on the requirements of the situation. We suggest that this stable trend within individuals may further extend to situations outside of speech processing and may be constant throughout life. The implications for social communication and perception ought to be further investigated.

### Supplementary Information

Supplementary Figures.

## Data Availability

The datasets generated and/or analyzed during the current study are available in the OSF repository, https://osf.io/5fzha/. For privacy reasons, the videos cannot be made public.
