# Supplementary material for: Stable eye versus mouth preference in a live speech-processing task
Source: Sci Rep. 2023 Aug 8;13:12878. doi: 10.1038/s41598-023-40017-8 (PMC10409748; doi:10.1038/s41598-023-40017-8)
Supplement: Supplementary file 1 — Supplementary Figures. [file 41598_2023_40017_MOESM1_ESM.docx]

Supplementary Information

**Stable eye versus mouth preference in a live speech-processing task**

Charlotte Viktorsson^1*^, Niilo V. Valtakari^2*^, Terje Falck-Ytter^1,3^, Ignace T. C. Hooge^2^, Maja Rudling^1^, Roy S. Hessels^2^

**Affiliations**

^1^Development and Neurodiversity Lab, Department of Psychology, Uppsala University; Uppsala, Sweden

^2^Experimental Psychology, Helmholtz Institute, Utrecht University, Utrecht, the Netherlands

^3^Center of Neurodevelopmental Disorders (KIND), Division of Neuropsychiatry, Department of Women’s and Children’s Health, Karolinska Institutet; Stockholm, Sweden

**S1**. Complete lists of sentences. Part A was read during the learning phase (the order of English and Finnish parts was counterbalanced). Part B was read during the recall phase.

**Part A.**

English

1. The boy has already left

2. There was a parade yesterday

3. She was very old

4. The cat jumped over the fence

5. Plants need water to grow

6. It’s already late

7. He made a cake for the party

8. She is my younger sister

9. Elephants are such kind creatures

10. I’m going to see a movie

11. It’s likely to rain

12. That book is amazing

13. Tomorrow they’ll go to the museum

14. She is easily offended

15. Today is a great day

16. Let’s run there together

17. They made dinner together

18. She dressed for the occasion

19. My father is a poor singer

20. My aunt is a doctor

21. Seven birds flew in the sky

22. That girl runs fast

23. Those clothes are too big

24. The dog went into the water

25. We’ll paint the house yellow

Finnish

1. Poika on jo lähtenyt

2. Paraati oli jo eilen

3. Hän oli vanha

4. Kissa hyppäsi yli

5. Kasvit tarvii vettä

6. Nyt on jo myöhä

7. Hän valmisti kakun juhliin

8. Hän on nuorin siskoni

9. Norsut ovat niin kilttejä

10. Minä menen elokuviin

11. Kohta voi sataa

12. Se on hyvä kirja

13. Huomenna he menevät museoon

14. Tyttö loukkaantuu helposti

15. Nyt on hieno päivä

16. Juostaan sinne kaksin

17. He valmistivat ruokaa

18. Hän pukeutui tilaisuuteen

19. Isäni ei osaa laulaa

20. Täti on lääkäri

21. Linnut lensivät ilmaan

22. Tyttö juoksee

23. Vaatteet ei mahdu

24. Koira meni veteen uimaan

25. Talo keltaiseksi

**Part B.**

1. It’s already late
2. That book is amazing
3. Nyt on jo myöhä
4. Aurinko paistaa tänään
5. She dressed for the occasion
6. Huomenna he menevät museoon
7. The sun is shining today
8. He likes open spaces
9. Kasvit tarvii vettä
10. The library is full of books
11. He made a cake for the party
12. Kirjastossa on kirjoja
13. The cat jumped over the fence
14. Isäni ei osaa laulaa
15. They’ll paint the house red
16. Kissa hyppäsi yli
17. My father is a poor singer
18. There will be a parade tomorrow
19. Norsut ovat niin kilttejä
20. Huomenna siellä on paraati
21. I’m going to see a movie
22. Plants need water to grow
23. Hän on nuorin siskoni
24. It’s likely to snow
25. They made dinner together
26. Poika on jo lähtenyt
27. Paraati oli jo eilen
28. Punainen talo
29. Canada is big with lots of people
30. Hän korjaa hampaita
31. Juostaan sinne kaksin
32. Pidän aukeudesta
33. It’s likely to rain
34. Tyttö loukkaantuu helposti
35. She is easily offended
36. Let’s run there together
37. Kanadaan mahtuu paljon kaikkea
38. Hän oli vanha
39. Seven birds flew in the sky
40. Hän on vanhin veljeni
41. Me menemme teatteriin huomenna
42. My niece is a dentist
43. Tyttö juoksee
44. Talo keltaiseksi
45. He is my older brother
46. Täti on lääkäri
47. That girl runs fast
48. Voi sataa lunta
49. Tomorrow we’ll go to the theater
50. Today is a great day


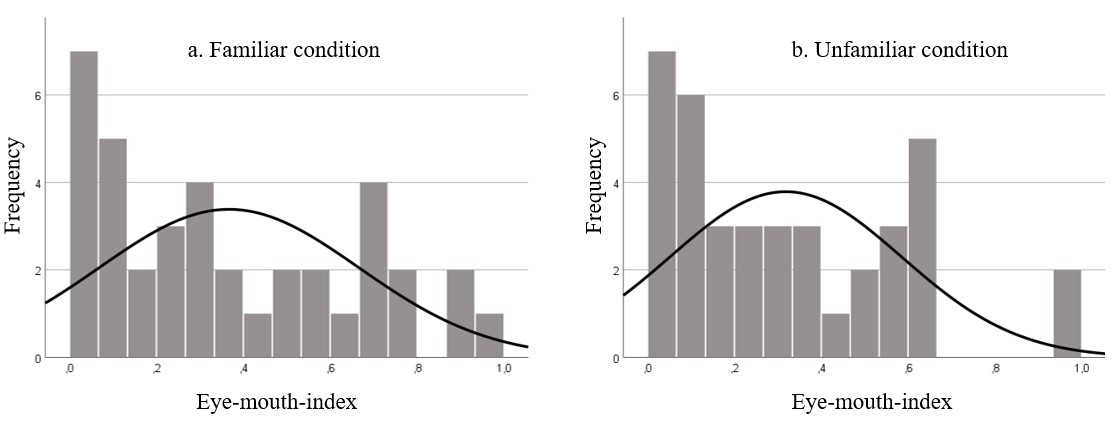


**Figure S1.** Distributional plots of the EMI in the Familiar (English) and the Unfamiliar (Finnish) condition.


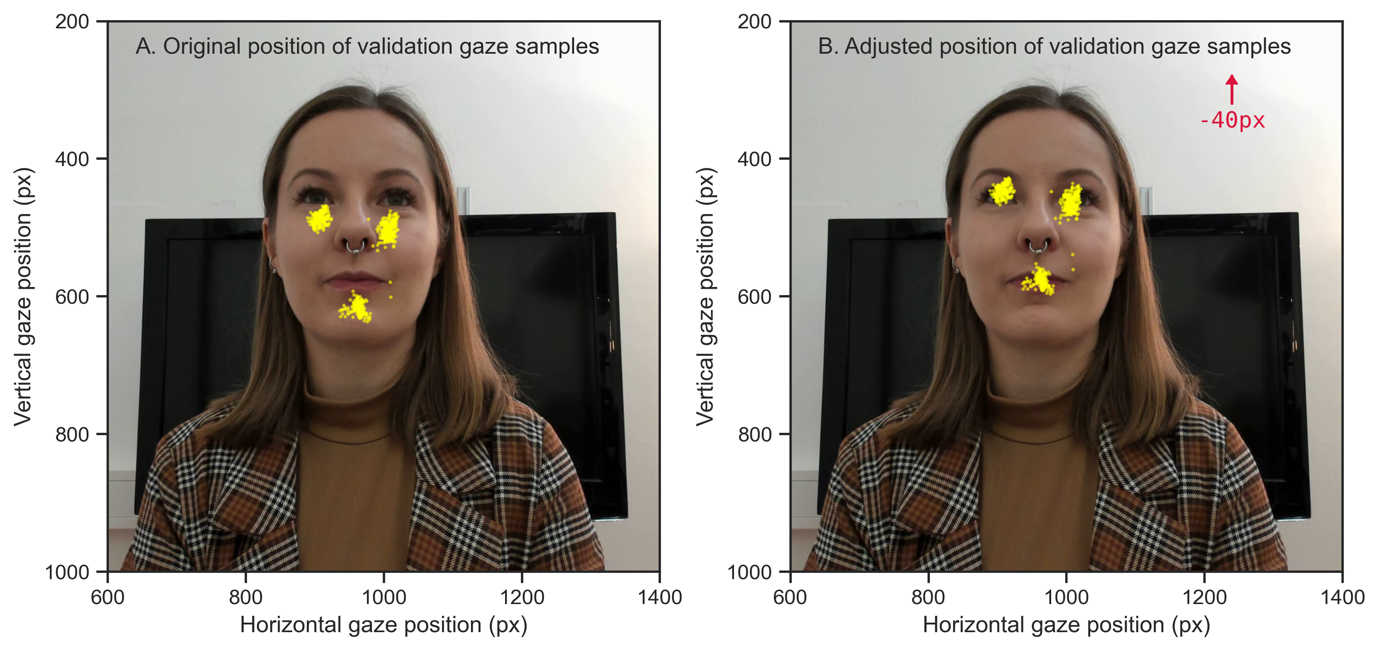


**Figure S2.** An illustration of the parallax error adjustment procedure for the 27 participants that had completed the additional validation procedure at the end of the experiment. Panel A depicts all gaze samples from the validation procedure for one example participant superimposed on the scene camera image in their original position. As can be seen, there is a positive offset in the gaze position on the vertical axis, but not on the horizontal axis. Panel B represents the gaze samples after they have manually been adjusted to match the position of experimenter’s eyes and mouth as they appear in the scene camera image. The red arrow and its accompanying text represent the direction and magnitude of the correction.


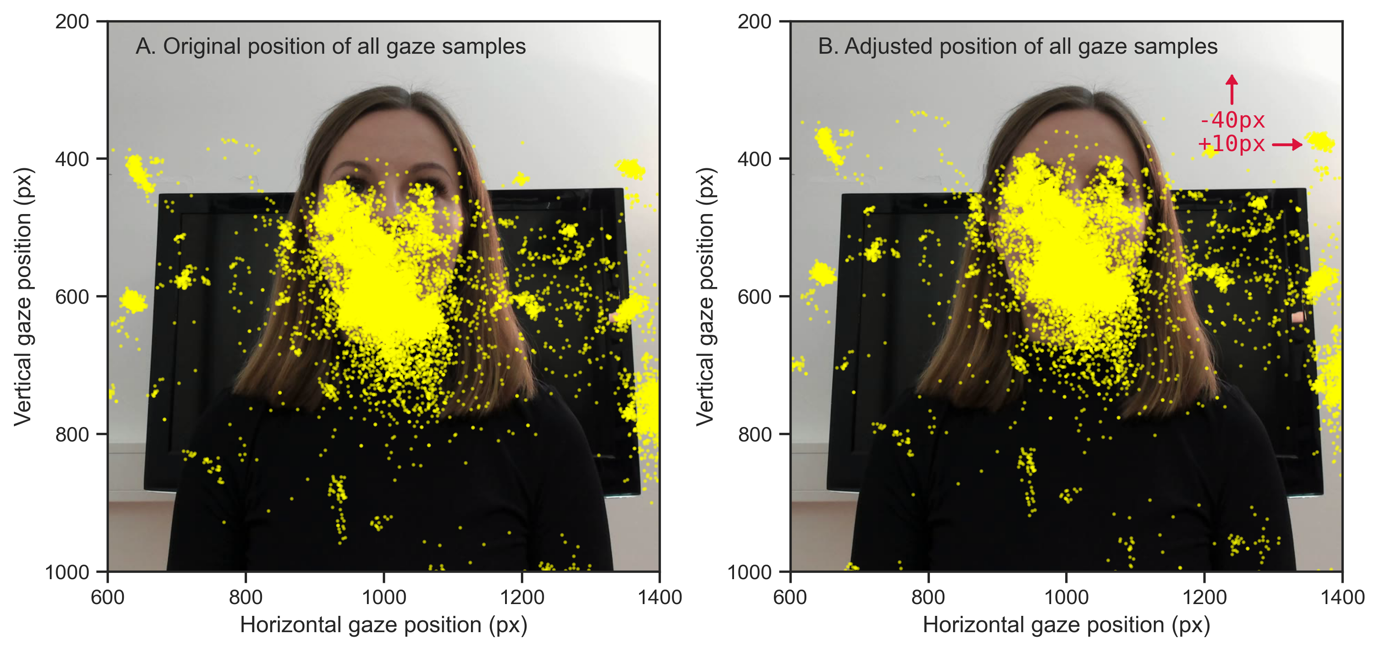


**Figure S3.** An illustration of the parallax error adjustment procedure for the 14 participants who did not complete the additional validation procedure. Panel A represents all gaze samples for both conditions for one example participant superimposed on the scene camera image in their original position. There appears to be a relatively large positive offset in the vertical gaze position signal and a relatively small negative offset in the vertical gaze position signal. Panel B represents the gaze samples after they have manually been adjusted to match the position of experimenter’s face as it appears in the scene camera image, factoring in the changes in head orientation due to looking down at the lists of sentences. The red arrows and their accompanying texts represent the direction and magnitude of the corrections.
